# Feta cheese proteins: Manifesting the identity of Greece׳s National Treasure

**DOI:** 10.1016/j.dib.2018.06.084

**Published:** 2018-06-28

**Authors:** Athanasios K. Anagnostopoulos, George Th. Tsangaris

**Affiliations:** Proteomics Research Unit, Center of Basic Research II, Biomedical Research Foundation of the Academy of Athens, Athens, Greece

## Abstract

Over the last years, there has been tremendous debate regarding the identity of feta cheese and under which terms such food with historical ties to ancient Greece can be discriminated among others, based on its unique traits and characteristics. This analysis sets the foundation towards a much anticipated control procedure, by deciphering for the first time the core elements of this food; its proteins. In this initial report, we amassed representative feta cheese samples/types from parts of Greece entitled to produce this “protected designation of origin” (p.d.o) cheese type and analyzed in full their protein content by employing exhaustive deep-proteome analyses. Several groups of proteins were identified, implicated in diverse functions as well as proteins under multiple abundances, while the final feta cheese protein list was set to include solely core-proteins identified in every analyzed sample. Through this data article we report, for the first time, the complete protein content of feta cheese, consisting of 489 proteins, thus setting the foundation towards developing a method for identification of the original Greek product.

**Specifications Table**

**Table:** 

Subject area	*Proteins, LC–MS/MS, cheese, food, proteomics*
More specific subject area	*Proteome cheese, feta cheese*
Type of data	*Excel file, Figures*
How data was acquired	1D-nanoLC–MS/MS, bottom-up proteomics
	Dionex Ultimate 3000 nanoHPLC system coupled to an LTQ Velos *Orbitrap Elite mass spectrometer* (Thermo Scientific, Rockford, IL, USA)
	PepMap^®^ RSLC, C18, 100 Å, 3-μm-bead-packed 15-cm column and 2-μm-bead-packed 50-cm column (Thermo Scientific)
	Proteome Discoverer 1.4 software (Thermo Scientific), Sequest search engine searching the *Rumintae*, *Ovis aries*.fasta databases.
Data format	*Analyzed*
Experimental factors	*Feta cheese samples were collected and systematically analyzed to fully characterize the protein content of this special cheese product*
Experimental features	*Whole-cheese analysis, casein-free sample analysis*
Data source location	*Athens, Greece*
Data accessibility	*Datasets are directly provided with this article*

**Value of the data**

• The identity of Feta cheese and the terms under which such a unique-type food can be distinguished from others still remains elusive.
• Through analysis of representative Feta cheese samples/types from all parts of Greece entitled to produce this “protected designation of origin” (p.d.o) cheese type, we report for the first time the complete list of Feta cheese proteins.
• The final list of feta cheese proteins consists of 489 distinct single-gene products.
• This dataset of Feta cheese proteins provides a step towards developing a method for identification of the original Greek product.

## 1 Data

**Fig. 1:**
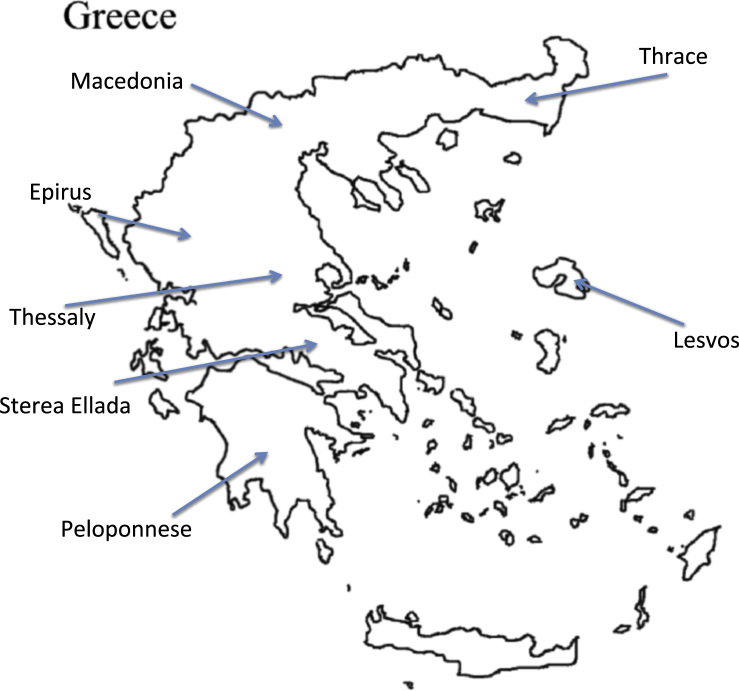
Geographical areas of Greece from which commercially available feta cheese samples were collected and analyzed. All feta-producing areas determined as p.d.o form the E.U. were selected and cheese samples thereof were included in the analysis.

**Fig. 2:**
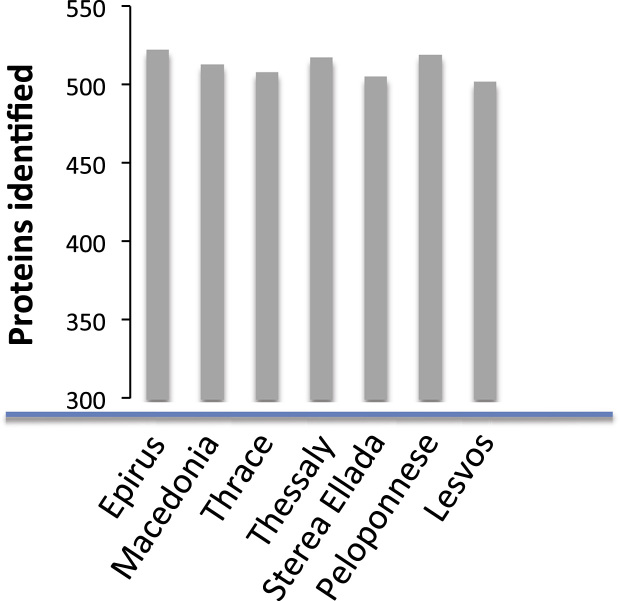
Variation of protein identification rates in correlation to the areas of feta cheese origin.

To unearth the set of proteins most representative for this type of material, as well as abolish any regional effects concerning the feta cheese samples, the final list of proteins was set to include molecules present in the entity of all analyzed samples. Feta cheese samples were collected from regions across Greece; in specific the seven areas depicted as p.d.o-producing from the EU (Fig. 1). In total, 25 commercially available feta samples were collected and analyzed. The identification procedure consisted of analysis of both full-casein and casein-free (including a casein removal step in the preparation process) samples. Altogether 489 proteins are included in the final list of proteins (Supplementary Table 1). How protein identification numbers varied across samples from different regions of collection is presented in Fig. 2 (total number of identified proteins: 500±17). We need to stress, once more, that incomplete databases regarding the species of both *Ovis aries* as well as *Capra hircus* add difficulty to this type of studies.

## 2 Experimental design, materials and methods

### 2.1 Sample collection

Representative commercially available feta cheese samples (25 samples in total) originating from relevant Greek areas designated as p.d.o form the E.U. were collected and immediately analyzed, without intervention of any freeze-thaw cycles.

### 2.2 Sample preparation

Cheese samples were powdered and 300 mg for each sample was suspended in 2 ml of ddΗ_2_Ο. Powdered feta cheese was sonicated for 36 s at 40% power with an ultrasonic homogenizer. Complete homogenization took place in a mixer at 1500 rpm for 1 h at 37 °C. Solutions were centrifuged at 10,000×*g* at 20 °C for 10 min to precipitate caseins from samples. Supernatants were removed and immediately subjected to analysis. Raw samples and depleted-casein samples were solubilized with urea sample buffer [1], and quantified with the Bradford assay [2]. Sample volumes corresponding to 200 ng of total proteins were further subjected to nanoHPLC–MS/MS.

### 2.3 Peptide generation and 1-D nanoLC–MS/MS analysis

Protein extraction form samples and generation of peptides, was performed exactly as described by our group elsewhere [3].

### 2.4 LC–MS/MS analysis

A modified protocol was followed in order to enhance identification rate of extracted peptides. LC–MS/MS analysis was performed exactly as described by us previously [4], [5].

The raw data files obtained by the Orbitrap mass analyzer were analyzed using the Proteome Discoverer software (Thermo Scientific), using the Sequest search engine applying the *Ruminantae* and *Ovis aries*.fasta databases.

## Appendix A Supplementary material

Supplementary material **Table 1:** List of core feta cheese proteins present in all samples analyzed. ^a^Accession number as appearing in the Swiss-Prot/UniprotKB database. ^b^Description (Protein name) as appearing in the Swiss-Prot/UniprotKB database. ^c^Sequence coverage=(number of the identified residues/total number of amino acid residues in the protein sequence)×100%. ^d^Number of identified proteins/protein groups. ^e^Number of unique peptides identified per protein/protein group. ^f^ Based on the calculation using the Proteome Discoverer software. ^g^Based on the calculation using the Proteome Discoverer software.
