# Supplementary material for: Feta cheese proteins: Manifesting the identity of Greece׳s National Treasure
Source: Data Brief. 2018 Jun 28;19:2037–40. doi: 10.1016/j.dib.2018.06.084 (PMC6141374; doi:10.1016/j.dib.2018.06.084)
Supplement: Supplementary file 1 — Supplementary material [file mmc1.docx]

The Authors Declare that they reserve their legal rights regarding the commercial application/use of the presented results. Relevant patent filed.
